# Retraction: Put the control back in the control condition: are brown, pink, and white noise neutral control stimuli?

**DOI:** 10.3389/fnins.2025.1720154

**Published:** 2025-10-13

**Authors:** Frontiers Editorial Office

The journal retracts the 25 April 2025 article cited above.

Following publication, the authors contacted the Editorial Office to request the retraction of the cited article, detailing concerns with the methodology. Specifically, a retrospective spectral analysis of the three types of noises uncovered that the stimuli contained only the lower frequency range from 0Hz up to 15kHz (for pink noise only up to 10kHz), but not higher than this. The conclusions reported in the article are therefore no longer supported by the data, so the article has been retracted.

The authors are invited to submit a new manuscript for peer review once these concerns have been addressed.

This retraction was approved by the Chief Editors of Frontiers in Neuroscience and the Chief Executive Editor of Frontiers. The authors agree to this retraction.

